# Exploring MiR-484 Regulation by *Polyalthia longifolia*: A Promising Biomarker and Therapeutic Target in Cervical Cancer through Integrated Bioinformatics and an In Vitro Analysis

**DOI:** 10.3390/biomedicines12040909

**Published:** 2024-04-19

**Authors:** Jiaojiao Niu, Yeng Chen, Hwa Chia Chai, Sreenivasan Sasidharan

**Affiliations:** 1 Institute for Research in Molecular Medicine (INFORMM), Universiti Sains Malaysia, Gelugor 11800, Pulau Pinang, Malaysia; niujiaojiao1987@student.usm.my; 2 School of Biological Engineering, Xinxiang University, Xinxiang 453003, China; 3 Department of Oral & Craniofacial Sciences, Faculty of Dentistry, Universiti Malaya, Kuala Lumpur 50603, Malaysia; 4 Department of Biomedical Science, Faculty of Medicine, Universiti Malaya, Kuala Lumpur 50603, Malaysia

**Keywords:** cervical cancer, miR-484, proliferation, prognostic genes, immunotherapy

## Abstract

Background: MiR-484, implicated in various carcinomas, holds promise as a prognostic marker, yet its relevance to cervical cancer (CC) remains unclear. Our prior study demonstrated the *Polyalthia longifolia* downregulation of miR-484, inhibiting HeLa cells. This study investigates miR-484’s potential as a biomarker and therapeutic target in CC through integrated bioinformatics and an in vitro analysis. Methods: MiR-484 levels were analyzed across cancers, including CC, from The Cancer Genome Atlas. The limma R package identified differentially expressed genes (DEGs) between high- and low-miR-484 CC cohorts. We assessed biological functions, tumor microenvironment (TME), immunotherapy, stemness, hypoxia, RNA methylation, and chemosensitivity differences. Prognostic genes relevant to miR-484 were identified through Cox regression and Kaplan–Meier analyses, and a prognostic model was captured via multivariate Cox regression. Single-cell RNA sequencing determined cell populations related to prognostic genes. qRT-PCR validated key genes, and the miR-484 effect on CC proliferation was assessed via an MTT assay. Results: MiR-484 was upregulated in most tumors, including CC, with DEGs enriched in skin development, PI3K signaling, and immune processes. High miR-484 expression correlated with specific immune cell infiltration, hypoxia, and drug sensitivity. Prognostic genes identified were predominantly epidermal and stratified patients with CC into risk groups, with the low-risk group showing enhanced survival and immunotherapeutic responses. qRT-PCR confirmed FGFR3 upregulation in CC cells, and an miR-484 mimic reversed the *P. longifolia* inhibitory effect on HeLa proliferation. Conclusion: MiR-484 plays a crucial role in the CC progression and prognosis, suggesting its potential as a biomarker for targeted therapy.

## 1. Introduction

Cervical cancer (CC) is a highly prevalent form of malignancy in the female reproductive tract system. It holds the fourth position globally in terms of both morbidity and mortality rates. The two main histological subtypes of squamous cell carcinoma and adenocarcinoma (CESC) account for most CC cases. Apart from persistent infection caused by high-risk human papillomavirus (HPV), the initiation and progression of CC can also be influenced by epigenetic modifications, such as miRNAs. Currently, radical hysterectomy, chemotherapy, and radiotherapy are the conventional treatment approaches for CC. However, the effectiveness of these treatments is limited by drug resistance, recurrence, and metastasis, leading to suboptimal outcomes for patients. Advanced patients with CC now have access to personalized treatment options, such as targeted therapies and immunotherapies. However, these treatment modalities still fail to improve overall survival (OS) and disease-free survival (DFS) for patients with CC. Therefore, it is urgent to identify more reliable targets and biomarkers to improve the prognosis of patients with CC.

Integrating mathematical, statistical, and computational methodologies in bioinformatics technology has paved the way for a comprehensive understanding of the tumor genomics landscape. Through the careful examination of large-scale RNA sequencing of tumor tissues across diverse databases such as The Cancer Genome Atlas (TCGA) and Gene Expression Omnibus (GEO), plenty of molecular markers and immunotherapy targets have been identified [1]. Additionally, single-cell RNA sequencing (scRNA-seq) is a powerful technique that can unveil the heterogeneous cellular and molecular characteristics of numerous cells in tumors [2]. The integration of these two techniques facilitates a more precise and comprehensive analysis of the molecular mechanisms associated with cancer, ultimately establishing a foundation for personalized therapeutic approaches.

MicroRNAs (miRNAs) are endogenous small non-coding RNAs that play crucial roles in a wide range of pathways under both physiological and pathological conditions. These single-stranded RNAs can bind imperfectly to the 3′-UTR seed region of target mRNAs, thereby degrading mRNA at the post-transcriptional level or inhibiting protein translation. Since the discovery of the first miRNA (Lin-4) in 1993, an increasing amount of evidence has pointed toward the association between the dysregulation of miRNAs and the onset of diseases, including tumors [3]. Several miRNAs, including oncomiR miR-21 and oncosuppressor miR-23b, have significant involvement in the initiation, development, and metastasis of CC [4]. The aforementioned statements suggest that miRNAs hold promise as diagnostic markers for CC and can be targeted for molecular therapies [5].

MiR-484, a specific miRNA in humans, assumes crucial functions in both the onset and resolution of various diseases, particularly in carcinomas. Its involvement spans across multiple cancer-related processes, including proliferation, apoptosis, angiogenesis, metastasis, and drug resistance. The aberrant expression of miR-484 has the potential to function as a diagnostic, therapeutic, and prognostic marker in various tumor types [6]. For instance, El-Maraghy, et al. [7] discovered that circulating miR-484 was upregulated in HCV-mediated hepatocellular carcinoma and could serve as a diagnostic and staging index for liver cirrhosis and fibrosis. Lee, et al. [8] identified miR-484 as an oncogene in prostate cancer since it promoted cell viability by targeting PSMG1 and was inversely correlated with DFS [8]. Xue, et al. [9] indicated that the high expression of exosomal miR-484 was associated with lower survival rates and could function as a potential prognostic marker for lung adenocarcinoma. In addition, miR-484 could directly target MAP2, activate the ERK1/2 signaling pathway, and promote tumor stemness, along with an association with an unfavorable prognosis in glioblastoma [10]. Conversely, under-expressed miR-484 was found in gastric cancer [11,12], colorectal cancer [13,14], and pancreatic cancer [15]. These findings implied the intricate and multifaceted role of miR-484 in diverse cancers. In the context of CC, a previous study found that treatment with the polyphenol-rich *P. longifolia* could induce HeLa cell apoptosis by downregulating miR-484 [16]. However, the prognostic value of miR-484 and its underlying mechanisms in CC remain incompletely understood. To address this knowledge gap, our study utilized bioinformatic methods to comprehensively analyze the diagnostic and prognostic implication of miR-484 in CC, hoping to provide references for mining potential therapeutic targets.

## 2. Materials and Methods

### 2.1. Data Source

The RNA-seq data of miR-484 from the TCGA database were downloaded from the UCSC Xena project (https://portal.gdc.cancer.gov/ (accessed on 18 August 2023)). The RNA-seq data of CESC and clinicopathological features, including age, tumor grade, radiotherapy, and survival data, were also obtained from the platform; normal samples and tumor samples with incomplete clinical data were excluded, and 283 samples were retained for further analyses. The GSE44001 dataset (*n* = 300 samples) from the GEO database (https://www.ncbi.nlm.nih.gov/geo/ (accessed on 18 August 2023)) was used for gene expression profiling and a survival analysis in external validation. To ensure consistency, the ENSEMBL Gene ID was converted to the Gene Symbol ID. Genes with <50% expression in the samples were excluded. The human CC scRNA-seq dataset GSE168652 was obtained from the GEO database.

### 2.2. MiR-484 Expression in Pan-Cancers

To determine the miR-484 expression in pan-cancers, we explored the differential miR-484 expression between tumor and adjacent normal tissues across all TCGA tumors using the Wilcoxon rank-sum test. The statistical significance was annotated (* *p* < 0.05; ** *p* < 0.01; *** *p* < 0.001).

### 2.3. Clinical Value of miR-484 in CC

To assess the clinical significance of miR-484 in CC, we employed the Wilcoxon test to investigate the correlations between the miR-484 level and clinicopathological parameters (age, grade, radiation, and tumor stage). A *p*-value < 0.05 implied marked differences.

### 2.4. Differential Expression Analysis and Functional Implications in CC

Based on the quartile level of miR-484, the top 25% and the bottom 25% of the 283 CC samples in the TCGA database were defined as the high-miR-484-expression group and low-miR-484-expression group, respectively. Differentially expressed genes (DEGs) between the two subgroups were obtained using the ‘Limma’ package, with adjusted *p* < 0.05 and |log2-fold change (FC)| > 1 as the criteria. The volcano plots and heat maps of DEGs were visualized using the “ggplot2” package (v3.3.3). To illustrate the potential functions of DEG-related miR-484, we further conducted the Gene Ontology (GO) enrichment and Gene Set Enrichment Analysis (GSEA) using the ‘clusterProfiler’ package, with adjusted *p* < 0.05, FDR < 0.25, and |NES| > 1 as the threshold. Subsequently, we utilized the “Maftools” package to explore somatic mutation information among patients with CC.

### 2.5. Relationship between miR-484 Expression and Immune Landscape in Patients with CC

The CIBERSORT algorithm (http://CIBERSORT.stanford.edu/ (accessed on 18 August 2023)) was adopted to estimate the tumor infiltration of 22 immune cell types [17]. For a further analysis, the immune checkpoint (ICP) and human leukocyte antigen (HLA)-related gene expression matrix were extracted for the DEG analysis. Concurrently, mutation data in CC were obtained from UCSC Xena and visualized using the “maftools” package [18]. Waterfall plots of the top 15 genes with the highest mutation frequencies in two subgroups were generated.

### 2.6. Tumor Stemness

A total of 26 stemness gene sets were gathered from an online tool, StemChecker (http://stemchecker.sysbiolab.eu/ (accessed on 18 August 2023)) [19], which compiled a comprehensive and the latest collection of stemness signatures derived from various sources. Then, a single-sample gene set enrichment analysis (ssGSEA) was utilized to quantitatively assess the enrichment scores of these stemness gene sets via the GSVA R package (v1.34.0). The difference in the stemness degree between the two subgroups was compared using the Wilcoxon test.

### 2.7. Selection of m6A, m5C, and m1A Genes

A total of 54 known *m6A*, *m5C*, and *m1A* RNA methylated modulators were extracted from the literature, including 23 *m6A* regulators: *METTL3*, *METTL14*, *WTAP*, *KIAA1429*, *RBM15*, *RBM15B*, *CBLL1*, *ZC3H13*, *YTHDF1*, *YTHDF2*, *YTHDF3*, *YTHDC1*, *YTHDC2*, *HNRNPC*, *HNRNPA2B1*, *IGF2BP1*, *IGF2BP2*, *IGF2BP3*, *FMR1*, *ELAVL1*, *LRPPRC*, *FTO*, and *ALKBH5*; 21 *m5C* regulators: *DNMT3A*, *DNMT3B*, *DNMT1*, *TET1*, *TET2*, *TET3*, *TDG*, *MBD1*, *MBD2*, *MBD3*, *MBD4*, *MECP2*, *NEIL1*, *NTHL1*, *SMUG1*, *UHRF1*, *UHRF2*, *UNG*, *ZBTB4*, *ZBTB38*, and *ZBTB33*; and 10 *m1A* regulators: *TRMT10C*, *TRMT61B*, *TRMT6*, *TRMT61A*, *YTHDF1*, *YTHDF2*, *YTHDF3*, *YTHDC1*, and *ALKBH1*.

### 2.8. Prediction of the Sensitivity Response to Therapeutic Agents

To pinpoint potential drugs based on miR-484 expression, the “pRRophetic” R package was utilized to calculate the half-maximal inhibitory concentration (IC_50_) value of each drug from the Genomics of Drug Sensitivity in Cancer website (GDSC; https://www.cancerrxgene.org (accessed on 18 August 2023)).

### 2.9. Construction and Verification of the Risk Model

The survival data from the UCSC Xena database were retrieved for the prognostic analysis using the “survival” (v3.2-10) and “survminer” (v.0.4.9) packages. Then, the univariate Cox regression and Kaplan–Meier model were used to identify miR-484-associated DEGs with prognostic values (*p* < 0.05). Multivariate Cox regression (stepAIC) was subsequently adopted to uncover the candidates to construct a suitable signature. Afterward, the risk score of each patient was generated: risk score = Σβi × Expi, where β represents the risk coefficient and Exp refers to the expression of prognostic genes (i). According to the median risk score, patients with CC were classified into high-risk and low-risk cohorts, and their survival status was compared using the Kaplan–Meier analysis. The model stability and reliability were validated in GSE44001 datasets. The GSEA method was implemented to figure out the functionally enriched pathways via the clusterProfiler R package.

### 2.10. Construction of an miRNA-mRNA-TF Network and Processing of scRNA-Seq Data

The miRNAs related to prognostic genes were predicted by the target scan in the ‘multiMiR’ package (version 1.20.0) [20] and MiRDB database (https://mirdb.org/ (accessed on 10 December 2023)). Transcription factors (TFs) for miRNA were predicted via the TransmiR database (http://www.cuilab.cn/transmir (accessed on 10 December 2023)). Similarly, the TF-miRNA-mRNA network was constructed with Cytoscape software (v 3.9.1). In addition, after processing the scRNA-seq dataset GSE168652 via the ‘Seurat’ package (version 4.1.0) [21], cells with gene counts less than 200 or greater than 5000 and mitochondrial gene fragments greater than 10% were excluded. The retained cells were pooled into one gene expression count matrix. Subsequently, the count data were normalized and scaled using the NormalizeData and ScaleData functions within the Seurat package. After dimension reduction and cluster identification through the RunUMAP and FindClusters functions, distinct cell clusters were identified and annotated by the SingleR R package. The “FindAllMarkers” and “FindMarkers” functions were utilized for Wilcoxon tests between pairs of cell clusters. The ‘featureplot’ function was then applied for visualizing the level of a particular gene.

### 2.11. Relationship between the Risk Model and Immune Background

The CIBERSORT and ESTIMATE algorithms were utilized to estimate immune infiltration by risk stratification. The differential levels of ICPs, tumor mutation landscape, and HLA genes were observed to characterize immunotherapeutic responses between risk subgroups.

### 2.12. Cell Culture and qRT-PCR

Human normal cervical cell lines (ECT1/E6E7) and CC cell lines (SiHa, CaSki, HeLa) obtained from American Tissue Culture Collection (ATCC, Manassas, VA, USA) were cultured in Dulbecco-modified-Eagle medium (Sigma-Aldrich, St. Louis, MO, USA) plus 10% fetal bovine serum (FBS, Biological Industries) and 1% penicillin–streptomycin (Biological Industries) at 37 °C in a humidified 5% CO_2_ incubator. Three replicates were set for each group of cell lines with 70–90% confluence. Subsequently, total RNA was extracted from cell lines using the TRIzol reagent. cDNAs were synthesized through reverse transcription using the SureScript First-Strand cDNA Synthesis Kit. The reaction conditions for qRT-PCR included pre-denaturation at 95 °C for 1 min, and a total of 40 cycles of denaturation at 95 °C for 20 s, annealing at 55 °C for 20 s, and extension at 72 °C for 30 s. The relative expression of genes was computed using the $2^{-\Delta \Delta \mathrm{Ct}}$ method, with glyceraldehyde-3-phosphate dehydrogenase (GAPDH) as an internal reference gene. Information on primers is presented in Table 1.

### 2.13. Plant Collection, Extraction, and IC_50_ Determination

The leaves of *Polyalthia longifolia* were collected from trees in University Sains Malaysia (Gelugor, Pulau Pinang, Malaysia). The collected leaves were washed twice with running tap water and once with distilled water, and then air-dried on a laboratory benchtop at room temperature (23 °C ± 2) for three weeks. Subsequently, the dried leaves were ground into coarse powder using a blender and stored in a clean, labeled airtight glass container with a deoxidizer sachet. The air-dried powders of *P. longifolia* leaves (80.0 g) were weighed and immersed in 400 mL of methanol for seven days at room temperature (23 °C ± 2), with occasional stirring using a glass rod. The extract was subsequently decanted and filtered using muslin cloth and Whatman No. 1 filter paper. Moreover, the filtrate was further concentrated at 40 °C using a vacuumed Rotary Evaporator (Buchi, St. Gallen, Switzerland). The final content was poured onto a clean, pre-weighed glass Petri dish and placed in an oven at 60 °C until methanol was completely evaporated, resulting in a paste-like dry extract. The PLME IC_50_ concentration used to treat HeLa cells was 15.20 µg/mL, according to previous MTT cytotoxicity assays [22].

### 2.14. Transfection of miRNA Mimics

The Syn-hsa-miR-484 miScript miRNA mimic purchased from Qiagen was transfected into cells following the manufacturer’s protocol. The miRNA mimic was resuspended to achieve a final concentration of 20 µM. Next, 1 × 10^5^ HeLa cells were seeded in the medium in 24-well plates before a 24 h incubation at 37 °C in a humidified 5% CO_2_ incubator. Subsequently, 5 nM of each miRNA mimic was prepared by diluting the stock miRNA mimics in a 500 µL serum-free culture medium. Afterward, the diluted miRNA was added to 3 µL of a HiPerFect transfection reagent (Qiagen, Venlo, The Netherlands) before being mixed through vortexing. This mixture was then incubated for 10 min at 25 °C (room temperature) to allow the formation of transfection complexes. Subsequently, the 24-well plate was gently rotated and the transfection complexes were dripped onto HeLa cells to make the transfection complexes evenly distributed. HeLa cells containing the transfection complexes were further incubated for 24 h at 37 °C in a humidified 5% CO_2_ incubator.

### 2.15. MTT Cell Proliferation Assay of Transfected HeLa Cells

The colorimetric cell viability MTT assay (Mosmann 1983) was performed to measure the cell viability of HeLa cells. HeLa cells were transfected with miR-484 mimics before treatment with the IC_50_ concentration of the PLME extract for 24 h. The experimental groups were the miR-484-mimic-transfected and vehicle-treated HeLa cell; the miR-484-mimic-transfected and PLME-extract-treated HeLa cell; and PLME-extract-treated HeLa cells. Subsequently, the cell viability assay was conducted using the 3-(4, 5-dimethylthiazol-2-yl)-2,5-diphenyltetrazolium bromide (MTT, Sigma-Aldrich, St. Louis, MO, USA) assay [23]. The MTT reagent was prepared at 0.5% *w*/*v* in phosphate-buffered saline. HeLa cells in 24-well plates were treated with 100 µL of the prepared MTT reagent and incubated for 4 h. After incubation, the medium was discarded before the addition of 500 µL of DMSO to each well to dissolve the formazan crystals. Then, the plate was placed on a stirrer to fully dissolve the formazan crystals before the absorbance was read at 540 nm with a microplate reader (Molecular Devices Inc., San Jose, CA, USA).

$$\mathrm{Cell}\;\mathrm{viability}\;\mathrm{was}\;\mathrm{calculated}:\;\mathrm{Cell}\;\mathrm{viability}\;(\%)=\frac{\mathrm{sample}-\mathrm{blank}}{\mathrm{control}-\mathrm{blank}}\times \;100\%$$

### 2.16. Statistical Analyses

Statistical analyses were performed using R version 4.2 (https://www.r-project.org). Experimental data were presented as the mean ± SD and analyzed with SPSS software version 20. One-way ANOVA and Tukey’s test were employed for multiple comparisons, and pairwise comparisons were assessed through the Wilcoxon rank-sum test. The survival analysis was performed using Kaplan–Meier curves and the log-rank test. A *p*-value  <  0.05 implied statistical meaningfulness.

## 3. Results

### 3.1. MiR-484 Expression in Normal and Tumor Tissues in TCGA

**Figure 1:**
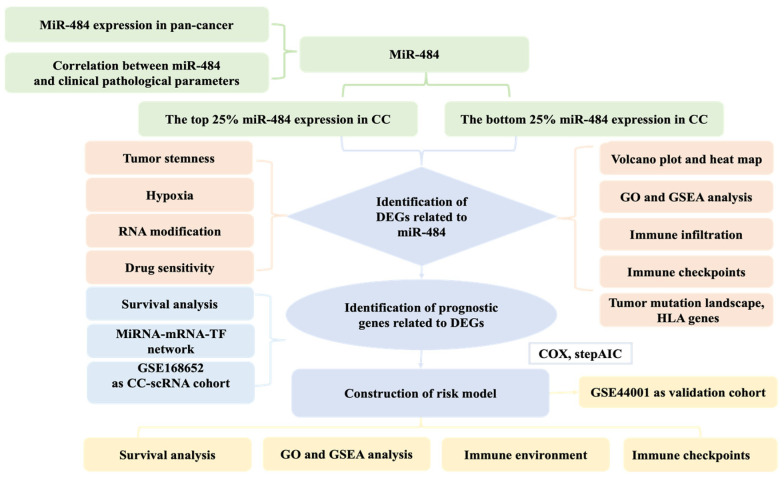
The detailed flow chart of this study.

**Figure 2:**
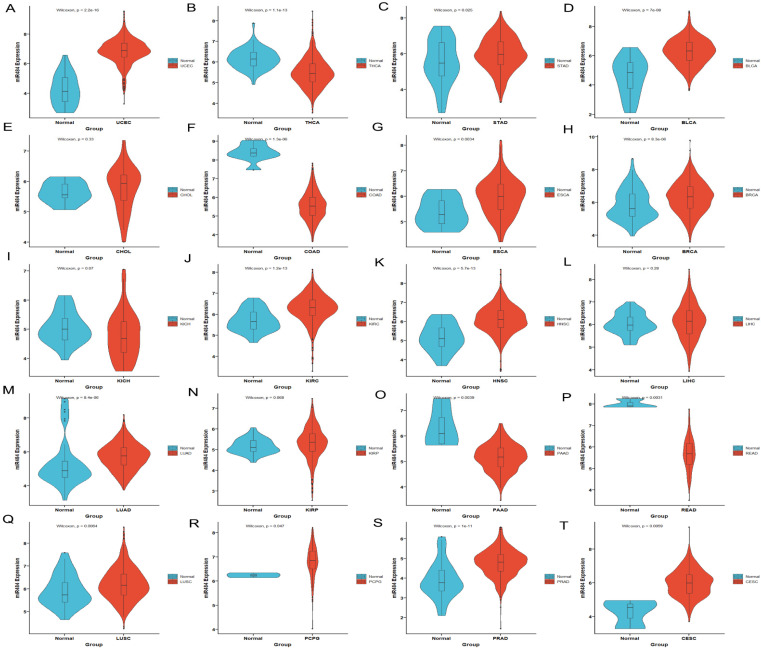
The analysis of miR-484 expression in pan-cancers. miR-484 expression is significantly upregulated in UCEC (*n* = 583) (**A**), STAD (*n* = 407) (**C**), BLCA (*n* = 430) (**D**), ESCA (*n* = 173) (**G**), BRCA (*n* = 1217) (**H**), KIRC (*n* = 607) (**J**), HNSC (*n* = 546) (**K**), LUAD (*n* = 585) (**M**), LUSC (*n* = 550) (**Q**), PCPG (*n* = 186) (**R**), PRAD (*n* = 551) (**S**), and CESC (*n* = 308) (**T**), while it is downregulated in THCA (*n* = 568) (**B**), COAD (*n* = 512) (**F**), PAAD (*n* = 182) (**O**), and READ (*n* = 177) (**P**). Moreover, there was no significant difference in the expression level of miR-484 among CHOL (**E**) (*n* = 45), KICH (**I**) (*n* = 89), HNSC (**L**) (*n* = 546) and KIRP (**N**) (*n* = 321). The value of ‘*n*’ represents the number of samples in each cancer type and ‘e’ represents exponent; *p* < 0.05 indicates statistical significance.

The detailed flow diagram is illustrated in Figure 1. MiR-484 expression in tumors and normal tissues was assessed using the TCGA and GTEx databases. As shown in Figure 2, miR-484 was overexpressed in 11 cancers, including stomach cancer (STAD), pheochromocytoma and paraganglioma (PCPG), lung squamous cell carcinoma (LUSC), lung adenocarcinoma (LUAD), kidney clear cell carcinoma (KIRC), head and neck cancer (HNSC), esophageal cancer (ESCA), breast cancer (BRCA), prostate cancer (PRAD), bladder cancer (BLCA), and endometrioid cancer (UCEC). In contrast, low miR-484 expression was observed in thyroid cancer (THCA), rectal cancer (READ), pancreatic cancer (PAAD), and colon cancer (COAD). Additionally, miR-484 expression was significantly elevated in CESC tissues from TCGA datasets compared to the corresponding controls.

### 3.2. MiR-484 Correlated Positively with TNM·T Stage

Correlations between miR-484 expression and clinicopathological parameters are shown in Supplementary Figure S1. MiR-484 expression was correlated positively with the TNM·T stage except T3. However, we did not observe any correlation between miR-484 expression and other clinicopathological parameters, including age, radiation, and tumor stage.

### 3.3. DEGs between High- and Low-miR-484 Groups in CC and Functional Enrichment Analysis

**Figure 3:**
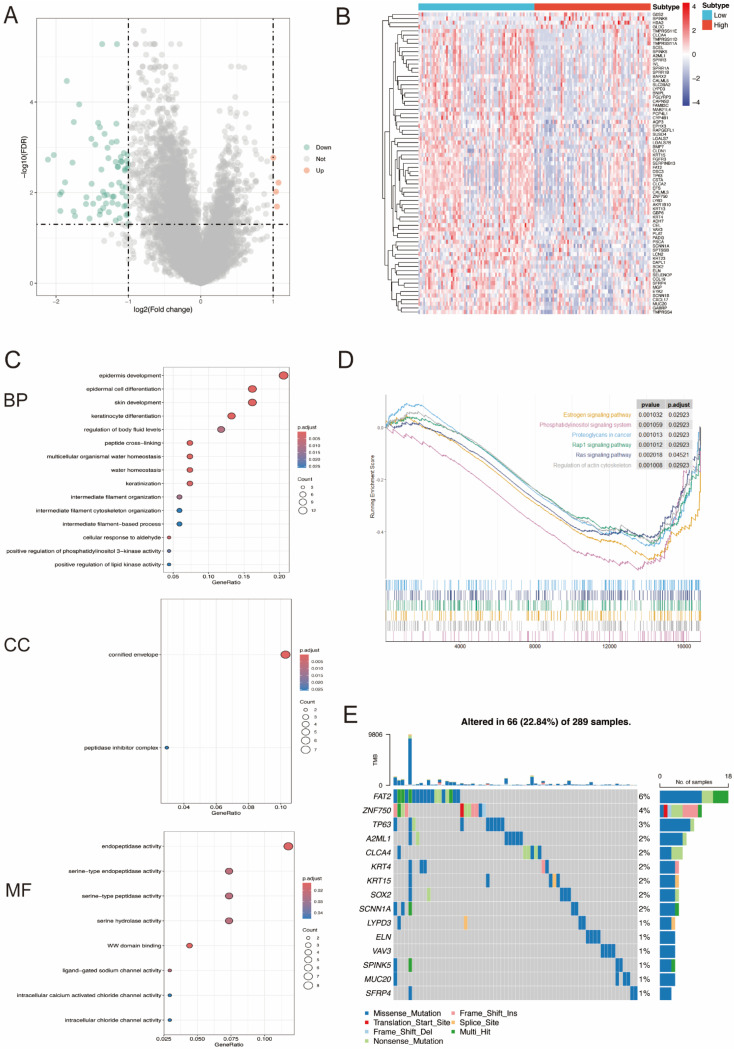
The analysis of miR-484-related DEGs. (**A**) The volcano plot shows 4 upregulated and 69 downregulated genes between high-miR-484 and low-miR-484 groups. The red and blue dots represent upregulated genes and downregulated genes, respectively. (**B**) The heatmap displays the expression level of 73 DEGs in each sample of different subgroups, with rows representing samples and columns representing DEGs. Samples were classified into the high-miR-484 and low-miR-484 groups, represented by red and blue bars in the figure, respectively. (**C**) A bar plot of GO enrichment in biological process terms, cellular component terms, and molecular function terms, respectively. (**D**) The gene set enrichment analysis (GSEA) of the altered signaling pathways in the 142 CC tissues based on the miR-484-associated DEGs. (**E**) The mutation landscape of miR-484-associated DEGs in CC tissues.

The Limma package was employed to pinpoint the DEGs linked to miR-484 in the TCGA-CESC dataset. Subsequently, the potential molecular mechanisms of these DEGs in CC were evaluated via clusterProfiler v4.8.3 software. The GO term analysis revealed that these DEGs were predominantly situated in the cornified envelope and peptidase inhibitor complex, participating in processes associated with skin, epidermis, and keratinocyte growth and differentiation. Their functions encompassed serine protease activity, WW binding, and involvement in multiple ion channel activities (Figure 3C). The GSEA results indicated that DEGs were primarily involved in biological pathways, such as the Ras signaling pathway, regulation of the actin cytoskeleton, Rap1 signaling pathway, estrogen signaling pathway, and phosphatidylinositol signaling system (Figure 3D). Somatic mutation results showed the top five genes (FAT2, ZNF750, TP63, A2ML1, and CLCA4) with the highest mutation frequencies among DEGs in CC tissues (Figure 3E).

### 3.4. MiR-484 Expression Correlated with the Infiltration of Immune Cells in CC Tissues

**Figure 4:**
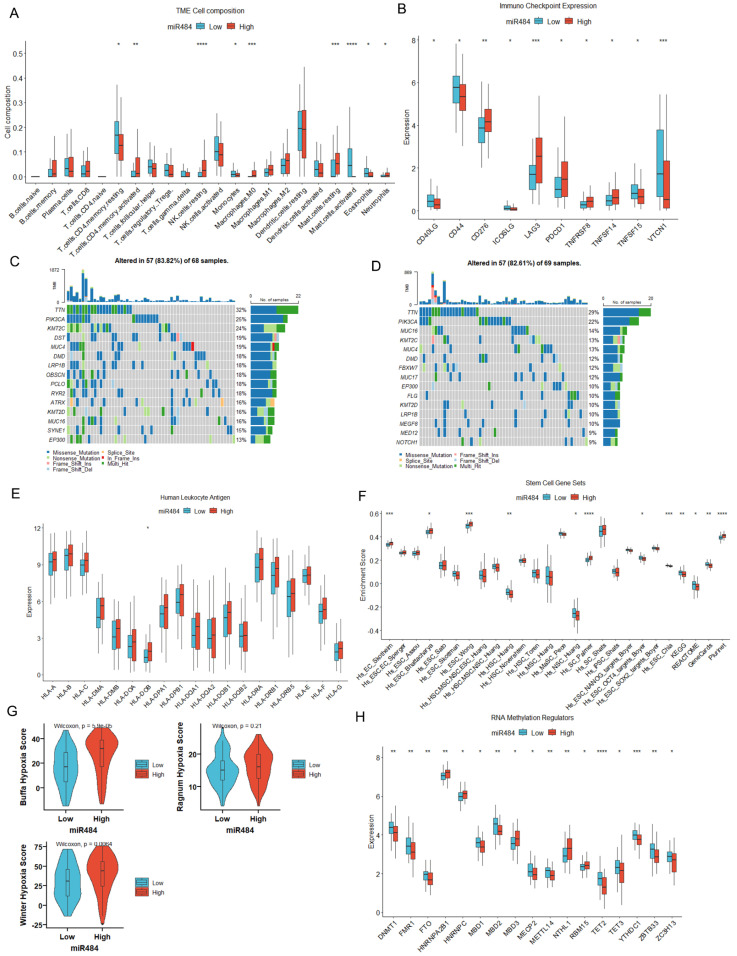
Analysis of immune landscape, tumor stemness, hypoxic landscape, and RNA modification in high- and low-miR-484 groups. (**A**) CIBERSORT algorithm showing differential infiltration levels of 22 immune cells between two subgroups. (**B**) Differential levels of ICPs between two subgroups in CC tissues. (**C**,**D**) Mutation landscape in high- and low-miR-484 groups of TCGA cohort. Differential levels of HLA family genes (**E**), tumor stemness genes (**F**), hypoxic scores (**G**), and RNA methylation regulators (**H**) between two subgroups in CC tissues (**** *p* < 0.0001; *** *p* < 0.001; ** *p* < 0.01; * *p* < 0.05).

The CIBERSORT algorithm was utilized to estimate the relative abundances of 22 immune cell types in the high- and low-miR-484 subgroups. As depicted in Figure 4A, miR-484 expression was positively correlated with the infiltration of activated memory CD4 T cells, resting NK cells, resting mast cells, M0 macrophages, and neutrophils. In contrast, miR-484 was negatively linked to the infiltration of resting memory CD4 T cells, activated mast cells, monocytes, and eosinophils.

### 3.5. MiR-484 Expression Correlated with ICPs, Mutation Landscape, and HLA Genes in CC

To comprehend the role of miR-484 in immunotherapy, we further probed into the relationship between miR-484 expression and ICPs. MiR-484 exhibited positive associations with CD276, LAG3, PDCD1, TNFRSF8, and TNFSF14, and negative associations with CD40LG, CD44, ICOSLG, TNFSF15, and VTCN1 (Figure 4B). Moreover, somatic mutation data from the TCGA database were utilized to discern the mutation landscape between the two subgroups. As illustrated in Figure 4C,D, the top four mutated genes within subgroups were TTN, PIK3CA, KMT2C, and MUC4. Furthermore, HLA is implicated in delivering antigen molecules during immunity and is essential in anti-tumor immunity [24]. Our study unveiled a marginally elevated expression of HLA genes in the high-miR-484 group compared to the low group (Figure 4E).

### 3.6. MiR-484 Expression Correlated with Tumor Stemness, Hypoxia, and RNA Modification

Cancer stem cells, characterized by their capacity for self-renewal, uncontrolled proliferation, and initiation of tumorigenesis, are recognized as the main contributors to the progression, metastasis, and treatment resistance of CC [25]. In our study, the ssGSEA algorithm was utilized to quantify the enrichment scores of the 26 stemness genes. The Wilcoxon test revealed significant enrichment of stemness genes, including Hs_EC_Skotheim, Hs_ESC_Bhattacharya, Hs_ESC_Wong, Hs_SC_Palmer, and Plurinet in the high-miR-484 group. Conversely, elevated levels of Hs_HSC_Huang, Hs_NSC_Huang, Hs_ESC_OCT4_targets_Boyer, and Hs_ESC_Chia were found in the low-miR-484 group (Figure 4F). Moreover, hypoxic conditions in the tumor microenvironment (TME) are widely acknowledged as a significant catalyst for CC progression [26,27]. In the current study, the Buffa Hypoxia Score, Ragnum Hypoxia Score, and Winter Hypoxia Score data obtained from the TCGA-CC cohort were utilized to evaluate the hypoxia landscape between miR-484 subgroups for patients with CC. The results confirmed a significant positive correlation between hypoxia scores and miR-484 expression (Figure 4G). Additionally, previous studies have proposed that RNA modifications, specifically m6A/m5C/m1A, contribute to the onset and progression of CC [28]. In this study, the levels of m6A modification genes HNRNPA2B1, HNRNPC, and RBM15, as well as m5C modification genes MBD3 and NTHL1, were markedly elevated in the high-miR-484 group. Conversely, m6A modification genes FMR1, FTO, and METTL14, and m5C modification genes DNMT1, MBD1, MBD2, MECP2, TET2, TET3, and ZBTB33, along with m1A modification genes YTHDC1 and ZC3H13, were significantly upregulated in the low-miR-484 group (Figure 4H).

### 3.7. Identification of Potential Drugs Targeting miR-484

**Figure 5:**
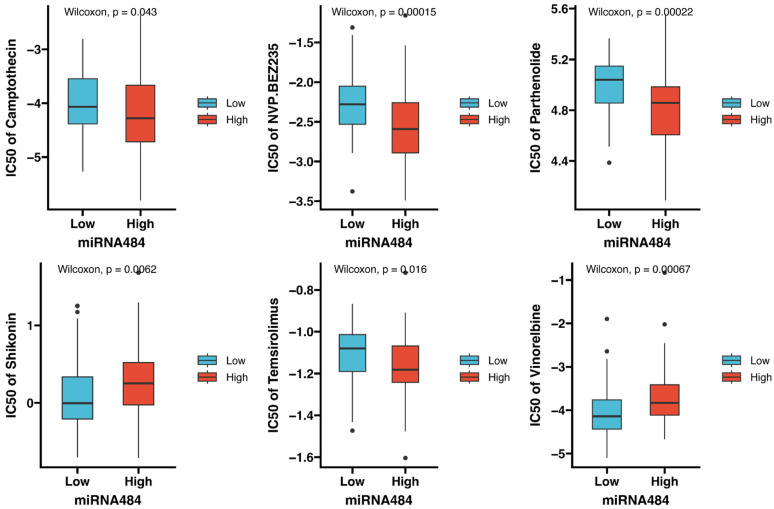
The box plots illustrate the IC_50_ values of six therapeutic agents between high- and low-miR-484-expression groups. Camptothecin, NVP.BEZ235, Parthenolide, and Temsirolimus exhibited lower IC_50_ values in the high-miR-484 group, while Shikonin and Vinorelbine exhibited lower IC_50_ values in the low-miR-484 group. *p* < 0.05 indicates statistical significance.

To ascertain potential drugs targeting miR-484 for patients with CC, the pRRophetic algorithm was used to assess the therapeutic response as per the IC_50_ in the GDSC database. In total, six compounds with significant differences in IC_50_ were identified between the high- and low-miR-484 groups. Camptothecin, NVP.BEZ235, Parthenolide, and Temsirolimus showed lower estimated IC_50_ values in the high-miR-484 group, suggesting that patients with high miR-484 expression might be more sensitive to these drugs. Conversely, Shikonin and Vinorelbine exhibited greater sensitivity in patients with lower miR-484 expression (Figure 5).

### 3.8. Construction and Verification of the Risk Model

**Figure 6:**
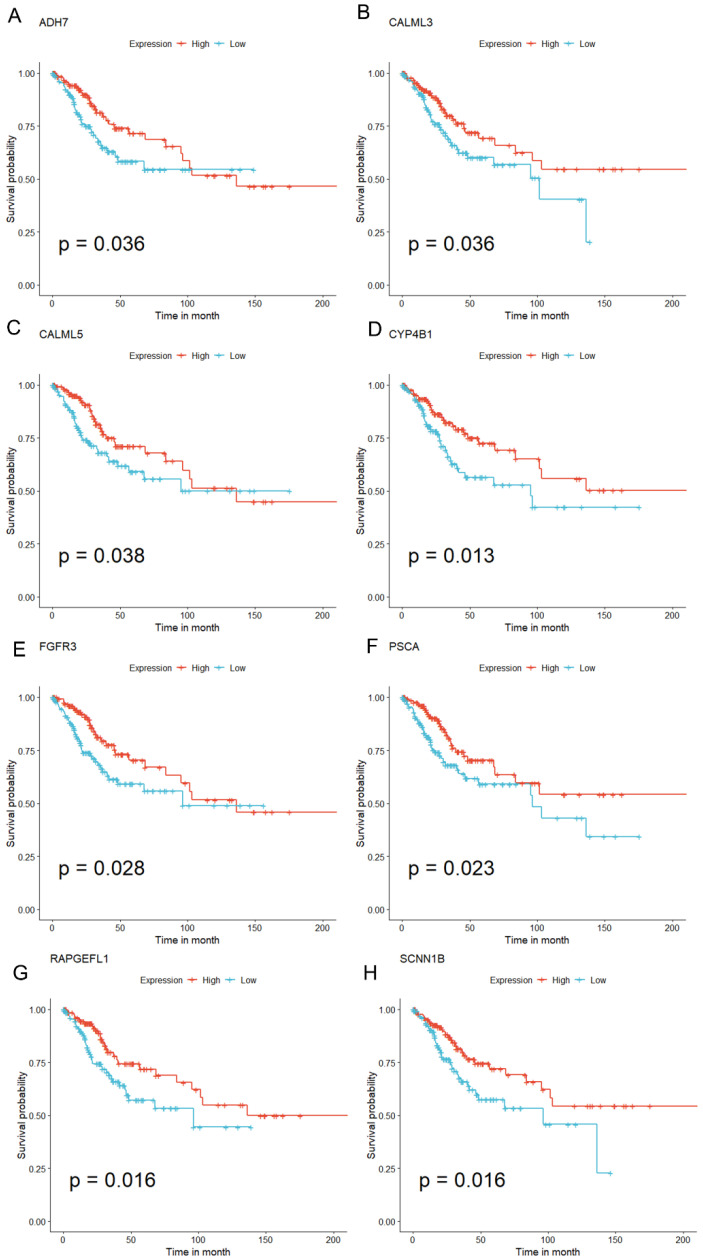
Associations between miR-484-DEG expression and OS outcomes based on the Kaplan–Meier models. Patients were categorized into high- and low-expression cohorts based on the median expression level of miR-484 DEGs. Patients had better OS with elevated expressions of ADH7 (**A**), CALML3 (**B**), CALML5 (**C**), AYP4B1 (**D**), FGFR3 (**E**), PSCA (**F**), RAPGEFL1 (**G**), and SCNN1B (**H**).

**Figure 7:**
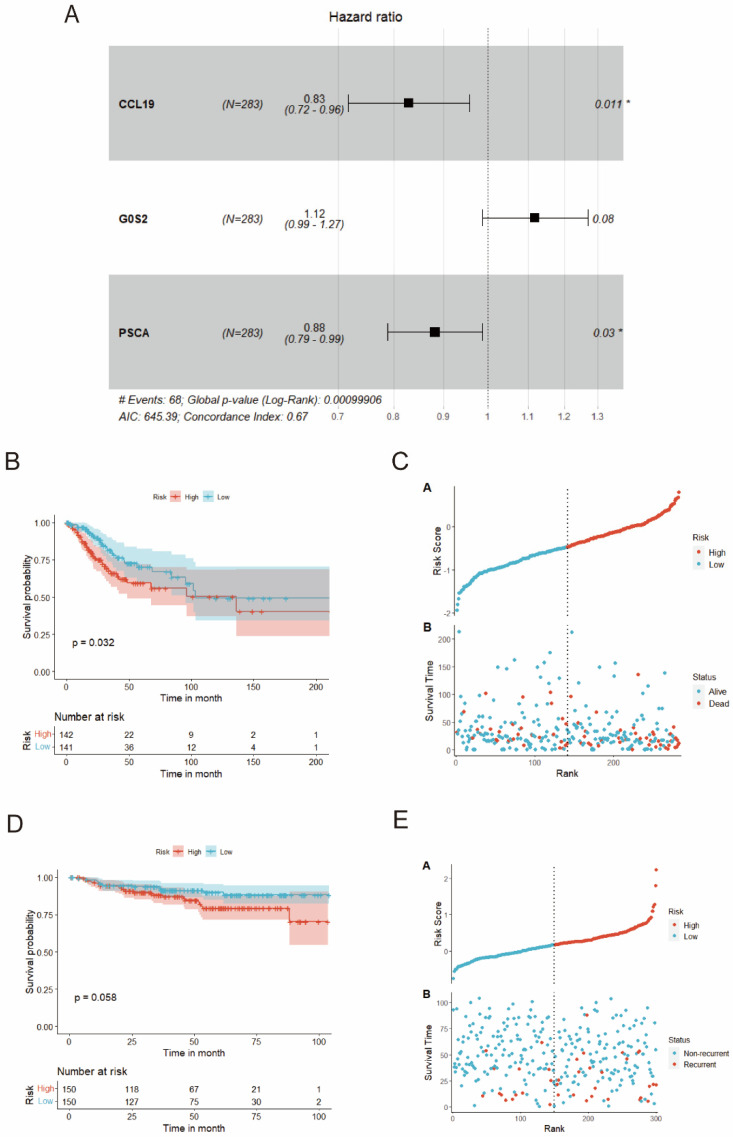
Construction and validation of miR-484-DEG-related prognostic models. (**A**) Among DEGs relevant to miR-484, three prognostic genes were screened by multivariate Cox analysis (stepAIC). (**B**) Kaplan–Meier survival curves showing OS outcomes according to relative high-risk and low-risk patients in TCGA-CESC data (*n* = 283). (**C**) Point plots for assessing risk distribution of patients between high- and low-risk groups from TCGA-CESC data. (**D**) Kaplan–Meier survival curves showing OS outcomes according to relative high-risk and low-risk patients in GSE44001 data (*n* = 300). (**E**) Point plots for assessing risk distribution of patients between high- and low-risk groups from GSE44001 data. * *p* < 0.05 indicates statistical significance.

To identify DEGs associated with the prognosis, eight genes were obtained via univariate Cox regression, namely ADH7, CALML3, CALML5, CYP4B1, FGFR3, PSCA, RAPGEFL1, and SCNN1B. Notably, the Kaplan–Meier model revealed that all these genes, which were downregulated by miR-484, could independently predict the outcome of patients with CC (*p* < 0.05). Patients in the TCGA-CESC cohort were categorized into high and low groups based on the median expression of each gene. The results indicated improved OS in the high group compared with the low group (Figure 6). Then, multivariate Cox regression (stepAIC) was employed to optimize the model, and three genes (CCL19, PSCA, and G0S2) were ultimately identified (Figure 7A). Furthermore, we calculated the risk score based on the levels of these genes: Risk score = (expression of CCL19 * (−0.187630321)) + (expression of PSCA * (−0.125610043)) + (expression of G0S2 * 0.112407253). Patients with CC were then stratified into high- or low-risk groups based on the median value of the risk score. The Kaplan–Meier analysis displayed that individuals in the high-risk group were more susceptible to shorter OS and higher mortality rates (Figure 7B,C, all *p* < 0.05). Furthermore, consistent results were obtained in the external validation dataset GSE44001, underscoring the precision of the risk model (Figure 7D,E, *p* < 0.05).

### 3.9. MiRNA-mRNA-TF Network and Cell Localization in scRNA-Seq Dataset for Prognostic Genes

**Figure 8:**
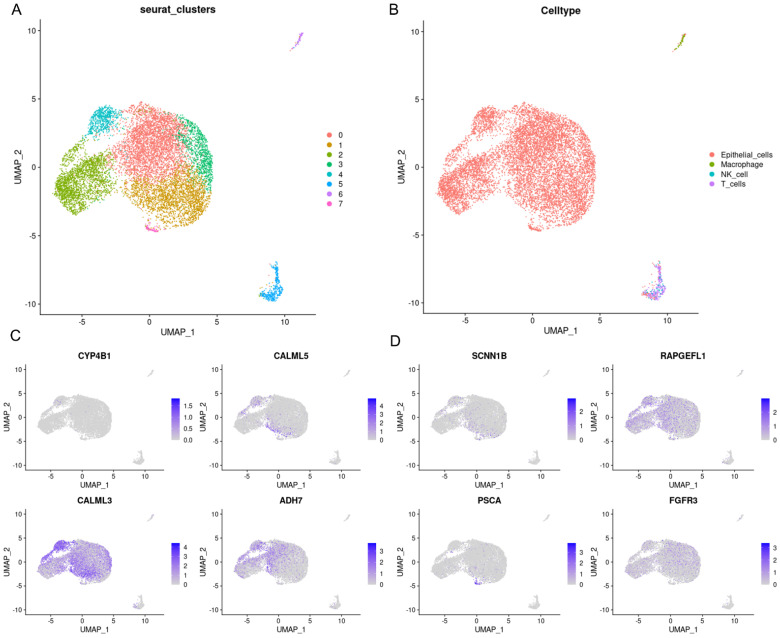
Single-cell sequencing analysis of GSE168652 and the cell localization of 8 prognostic genes associated with OS. (**A**) Cluster analysis and dimension reduction. All cells in GSE168652 were divided into 8 cell clusters. (**B**) The cells are categorized into epithelial cells, macrophages, NK cells, and T cells based on surface marker genes. (**C**,**D**) UMAP plots of the three prognostic genes.

To better understand the molecular regulatory mechanisms of prognostic genes, 8 prognostic genes and 64 miRNAs were integrated into the network. We discovered that FGFR3 was regulated by has-mir-99b-5p, while RAPGEFL1 was modulated by has-mir-27a-3p (Supplementary Figure S2A). Additionally, 8 prognostic genes and 39 TFs were merged into the network. We observed that FOXC1 and Jun were the most significantly connected with prognostic genes (Supplementary Figure S2B). Subsequently, we utilized the GSE168652 dataset to analyze the subcellular localization of eight prognostic genes in the TME. The GSE168652 dataset comprised eight cell populations and four major cell types, including epithelial cells, macrophages, NK cells, and T cells (Figure 8A,B). Notably, all these prognostic genes were highly expressed in epithelial cells, followed by macrophages and T cells (Figure 8C,D).

### 3.10. GSEA Analysis for DEGs between High- and Low-Risk Subgroups

**Figure 9:**
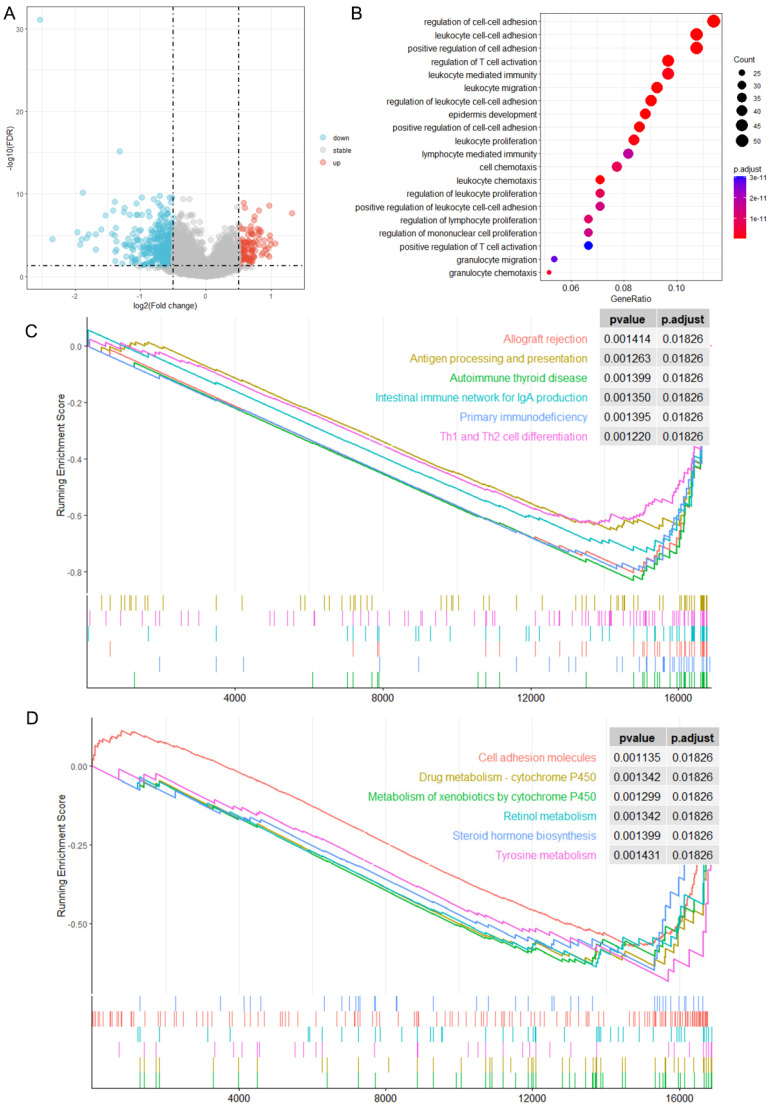
Enrichment-based assessments for DEGs linked with risk score. (**A**) Volcano plot showing DEGs associated with risk group and red/blue reflect up/downregulated genes, respectively. (**B**) GO enrichment of risk-score-related DEGs. (**C**,**D**) Gene set enrichment analysis (GSEA) identifying the altered signaling pathways of risk-score-related DEGs in CC.

DEGs between high- and low-risk subgroups were visualized in volcano plots (Figure 9A). The GO analysis revealed that DEGs linked to the risk model were mainly enriched in cell proliferation, adhesion, migration, and immunity (Figure 9B). The GSEA result demonstrated a higher enrichment of genes in immune-signaling pathways, including allograft rejection, antigen processing and presentation, autoimmune thyroid disease, the intestinal immune network for IgA production, primary immunodeficiency, and Th1 and Th2 cell differentiation. Additionally, tumor-related pathways including cell adhesion molecules, retinol metabolism, steroid hormone biosynthesis, tyrosine metabolism, and drug metabolism—cytochrome P450 were also implicated (Figure 9C,D).

### 3.11. The Immune Landscape and Immunotherapeutic Response of Prognostic Model

**Figure 10:**
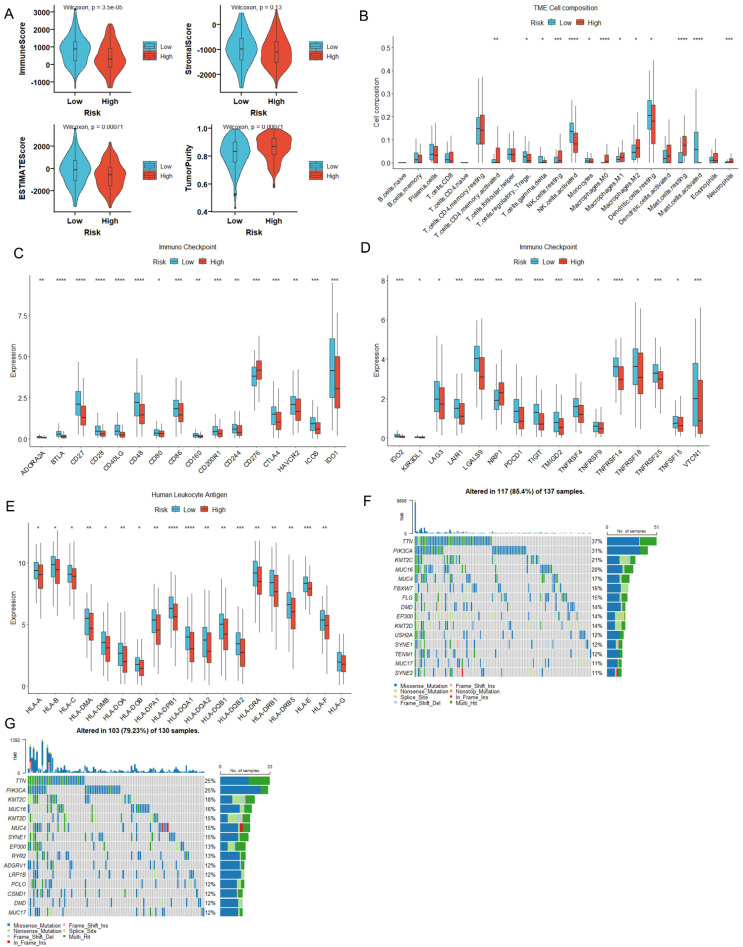
Immune landscape between high- and low-risk groups. (**A**) Differential expression of ESTIMATE score, immune score, stromal score, and tumor purity between two subgroups using ESTIMATE algorithm. (**B**) Differences in the infiltration levels of 22 immune cell types between two subgroups using CIBERSORT algorithm. Differential expression of ICP-related genes (**C**,**D**) and HLA genes (**E**) in the high- and low-risk groups. (**F**,**G**) Mutation landscape in the high- and low-risk groups of TCGA cohort (**** *p* < 0.0001; *** *p* < 0.001; ** *p* < 0.01; * *p* < 0.05).

To further elucidate the immune landscape between risk subtypes, we employed CIBERSORT and ESTIMATE algorithms to compare immune cell infiltration levels, stromal score, immune score, ESTIMATE score, and tumor purity. The obtained results revealed that the high-risk group exhibited a higher prevalence of M0, M1, and M2 macrophages, activated memory CD4 T cells, resting NK cells, resting mast cells, and neutrophils, along with higher tumor purity and lower immune scores. Conversely, the low-risk group, with higher immune scores and ESTIMATE scores, displayed more activated NK cells, activated mast cells, regulatory T cells (Tregs), gamma delta T cells, and resting dendritic cells (Figure 10A,B). In addition, most ICPs, particularly PDCD1 and CTLA4, were markedly downregulated in the high-risk group (Figure 10C,D). Furthermore, two additional immunotherapy indicators, HLA and Tumor Mutation Burden (TMB), were concurrently examined. All HLA genes were dramatically decreased in the high-risk group, implying immune dysfunction in the high-risk group (Figure 10E). In addition, the somatic mutation analysis indicated the top four genes (TTN, PIK3CA, KMT2C, and MUC16) with the highest mutation frequencies with the risk signature (Figure 10F,G). However, the TMB values did not differ between the two risk subgroups. Overall, these findings validate that low-risk individuals might have better outcomes when treated with immunotherapy.

### 3.12. qRT-PCR Validation for Key Prognostic Genes

**Figure 11:**
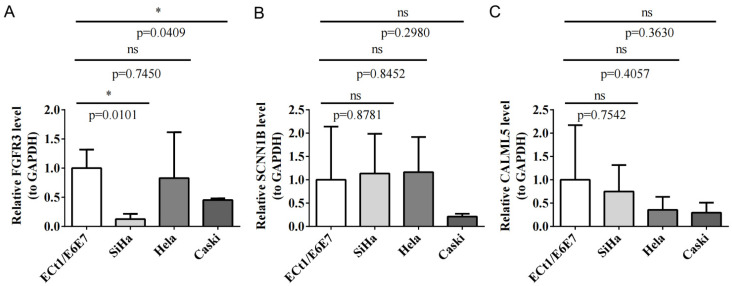
The expression of key prognostic genes was analyzed between tumor and normal cell lines using qRT-PCR through Student’s *t*-test. FGFR3 was significantly downregulated in SiHa and Caski cells compared to the normal cell line ECt1/E6E7. (**A**–**C**) represent FGFR3, SCNN1B, and CALML5, respectively. The data are expressed as the mean ± SD (*n* = 3), where * *p* ˂ 0.05 indicates statistical significance, ns = not significant.

To verify key prognostic gene expression, the qRT-PCR method showcased that FGFR3 was markedly downregulated in SiHa and Caski cells in contrast to control cells (*p* < 0.05, Figure 11A). SCNN1B expression was slightly lower in Caski cells (Figure 11B), while CALML5 was minorly downregulated in CC cell lines compared to normal cells (Figure 11C).

### 3.13. MiR-484 Increases the Survival of PLME-Treated HeLa Cells

**Figure 12:**
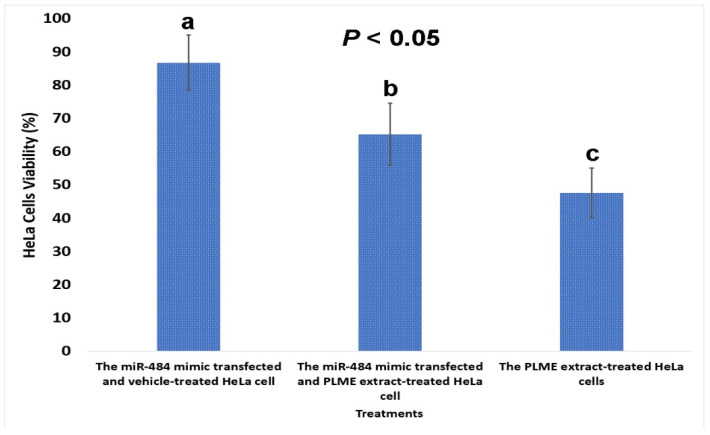
Cell viability of HeLa cells with different treatments by MTT assay. The data are expressed as mean ± SD (*n* = 3). Different letters (a–c) indicate significant differences (*p* < 0.05) using one-way ANOVA, followed by Tukey’s multiple comparison tests.

To delve into the underlying mechanism of miR-484 in HeLa cells, an in vitro survival assay was conducted using the MTT assay. As depicted in Figure 12, the percentage of viability (86.72 ± 0.13%) in HeLa cells transfected with the miR-484 mimic was significantly increased (*p* < 0.05), whereas the lowest proliferation (47.62 ± 0.01%) was observed in PLME-treated cells. The transfection of HeLa cells with the miR-484 mimic, along with PLME treatment, reduced cell viability to 65.23 ± 0.07%. These findings demonstrate that miR-484 has the potential to reverse the effects of PLME on HeLa cells, and increase cell viability by approximately 2.0 times compared to cells treated solely with PLME.

## 4. Discussion

CC is a prevalent gynecological tumor, with an unacceptably high fatality rate. Despite the considerable progress and improvements in the treatment, tumor recurrence and metastases emphasize the urgent need for new therapeutic approaches. The miRNA-targeted cancer therapy, particularly for CC treatment, has recently garnered considerable interest. MiR-484, one of the crucial miRNA regulators, has been observed to be dysregulated in various malignant tumors, indicating its potential as a potential molecular marker for cancer diagnoses and prognoses. Nevertheless, the prognostic value and potential mechanisms of miR-484 in CC are yet to be elucidated.

Based on current knowledge, this study marks a significant milestone in the field as it is the first report to comprehensively analyze the carcinogenic behavior and prognostic significance of miR-484 in CC by integrating bioinformatics and in vitro experiments. Our findings indicated an elevated miR-484 expression in CC compared to normal samples in the TCGA dataset.

To gain a deeper understanding of the potential mechanism of miR-484 in CC progression, we proceeded to perform GO and GSEA analyses to identify the biological pathways that are associated with DEGs. The GO analysis revealed that biological processes are primarily linked to the development and differentiation of the skin, epidermis, and keratinocyte, which suggested that these biological processes may have a significant impact on cancer-related events, such as cell differentiation and epithelial–mesenchymal transition (EMT). In addition, GSEA results showed that miR-484-related DEGs were mainly involved in the Ras signaling pathway, regulation of the actin cytoskeleton, Rap1 signaling pathway, Proteoglycans in cancer, estrogen signaling pathway, and phosphatidylinositol signaling system. In line with previous findings, the phosphatidylinositol 3-kinase/protein kinase-B (PI3K/AKT) pathway has been identified as a key driver in the carcinogenesis and metastasis of CC [4]. Notably, PI3K has been recognized as a significant factor in suppressing the Ras signaling pathway in CC. The simultaneous occurrence of Ras gene mutation and PI3K overexpression, along with oncogenic HPV, accelerates the progression of HPV-immortalized epithelial cells toward invasive tumors [29]. Furthermore, estrogen receptors hold significant importance in the female reproductive system. Extensive studies have shown that Genistein (Gen), a phytoestrogen, can stimulate the proliferation of human CC cells by activating the estrogen-receptor-mediated PI3K/Akt-NF-κB pathway. Subsequently, the actin cytoskeleton organization regulates the viability and invasion of cancer cells. The HPV16 E6-induced downregulation of NHERF1 has been reported to promote cytoskeleton assembly and cell invasion, thereby contributing to CC development [30]. Moreover, the ‘Rap1 signaling pathway’ [31] and ‘Proteoglycans in cancer’ [32] are associated with invasion and metastasis. These findings confirm the crucial role of miR-484 in the onset and advancement of CC by participating in various oncogenic pathways.

A previous study found that dysregulated miRNAs were linked to the initiation and progression of inflammation-triggered CC [33]. Our study unraveled a potential correlation between the miR-484 level and immune cell infiltration in CC. Specifically, the miR-484 level negatively correlated with activated mast cells and eosinophils while positively correlating with activated memory CD4 T cells, M0 macrophages, and neutrophils. CD4 cells are extensively recognized for their anti-tumor immune function in the TME. However, recent reports highlighted decreases in OS and DFS when the CD8/CD4 ratio < 2 [34]. Moreover, the reversal of the CD8/CD4 ratio in patients with CC was strongly associated with accelerated tumor growth and lymph node metastasis [35], which suggested a promoting tumor role of CD4 in the TME of CC. In addition, mast cells exhibited a negative correlation with the severity of dysplasia, declining from dysplasia to malignancy [36], and activated mast cells were inversely associated with the immune-related risk score in CC [37]. Furthermore, M0 macrophages could enhance tumor cell proliferation and invasion [38], and the communication between M0 macrophages and naïve CD4 T cells established an immunosuppressive microenvironment in CC [38]. Additionally, eosinophils were positively associated with favorable outcomes, whereas neutrophils were indicators of worse survival outcomes in CC [39]. In summary, these findings confirm the effectiveness of the model and provide valuable perspectives for tailored immunotherapy in CC.

The advent of ICP blockade therapy has recently brought about a revolutionary change in the treatment of CC [40]. Our study disclosed that patients with elevated miR-484 levels might potentially benefit from immunotherapies targeting classical ICPs, such as LAG3 and PDCD1. In addition, there is abundant evidence that specific genomic alterations are implicated in the initiation and progression of CC [41]. In our study, we investigated the tumor mutation landscape between the two subgroups and noted the top four mutated genes: TTN, PIK3CA, KMT2C, and MUC4. Similarly, a previous study has revealed somatic mutations in PIK3CA, TTN, and MUC4 in CC pathogenesis [41]. Hence, it is imperative to grasp the intricate interaction between miR-484 and somatic mutations to elucidate the complex molecular mechanisms underlying CC. Additionally, given the pivotal role of HLA genes in the immunotherapeutic responses, our study revealed a subtle positive correlation between the expression of all HLA genes and miR-484, implying that individuals with elevated miR-484 levels might be better suited to receive immunotherapy. Furthermore, in the context of CC, cancer stem cells, hypoxia, and RNA methylation significantly contribute to cell proliferation, metastasis, metabolism, and therapeutic resistance within the TME [25,26,27,42]. This current study identified significant differences in most stem cell markers and RNA methylation levels between the two subgroups. Moreover, elevated hypoxia scores were noted in the high-miR-484 subgroup, indicating the potential oncogenic role of miR-484 in CC.

The analysis of therapeutic agents targeting miR-484 inferred that patients with elevated miR-484 levels might experience favorable outcomes when treated with DNA-damage-repair-related drugs such as Camptothecin, PI3K/mTOR inhibitors such as NVP-BEZ235, NF-κB inhibitors such as Parthenolide, and mTOR inhibitors such as Temsirolimus. In contrast, patients with lower miR-484 levels might be more sensitive to Shikonin and Vinorelbine. Overall, our findings may provide more precise personalized treatment for patients with CC.

For the eight identified independent prognostic genes, FGFR3 and SCNN1B were downregulated in cancer cells and could function as protective prognostic indicators for CC [43,44,45]. ADH7, implicated in tumor progression by modulating the retinoic acid metabolic process, could predict favorable outcomes for CC [46]. CALML3 was recognized as a crucial marker and suppressor for liver cancer metastasis [47]. CALML5 was involved in the regulation of epidermal differentiation, and CALML5 silencing deteriorated cervical squamous cell carcinoma [48]. CYP4B1 was regarded as a prognostic biomarker and tumor suppressor for lung cancer (LC) [49], and missense variants in CYP4B1 were correlated with LC susceptibility [50]. RAPGEF1 encodes a member of the Ras family of small GTPases and exerts anticancer effects, which was associated with promoter hypermethylation in cervical squamous cell carcinoma [51]. PSCA was an auspicious biomarker for gastric cancer, and the downregulation of PSCA was associated with an enhanced proliferation and unfavorable prognosis [52]. The above findings suggested that these genes could serve as protective prognostic factors in carcinomas, in support of our results. Subsequently, we selected key prognostic genes for cellular experimental validation in CC. qRT-PCR results revealed a significant decrease in FGFR3 expression in SiHa and CaSki cells compared to control cells, consistent with previous findings [44]. CALML5 exhibited a minor downregulation in CC cell lines, and SCNN1B expression was slightly lowered in CaSki cells compared to the other three cell lines. Furthermore, we screened essential TFs and miRNAs that interacted with these genes and identified two pivotal TFs: FOXC1 and JUN. FOXC1 and JUN assumed critical roles in promoting cancer proliferation, metastasis, and chemoresistance [53,54]. Additionally, we discerned the predominant localization of prognostic genes in epithelial cells via scRNA-seq. According to previous reports, epithelial cell polarization is crucial in EMT [55]. In addition, the intricate progression of CC is influenced by the interaction between cancerous epithelial cells, stromal cells, and immune cells in the TME [56].

Subsequently, we constructed a risk model based on three genes, namely CCL19, PSCA, and G0S2. CCL19 was confirmed as a tumor suppressor in colorectal cancer [57] and gastric cancer [58], while it could enhance the malignancy of small-cell LC [59]. In our study, CCL19 was downregulated by miR-484 and functioned as a protective prognostic indicator for CC. G0S2 was identified as a prospective biomarker in glioblastoma stem-like cells [60], and the methylation-induced downregulation of G0S2 was linked to the inhibited invasion of IDH1-mutant glioblastoma cells and an improved prognosis [61]. Similarly, the oncogenic role of G0S2 was confirmed in the current study, as it served as an adverse prognostic index for CC. Moreover, the patients were stratified based on their median risk score. The high-risk population displayed an immunosuppressive phenotype characterized by high tumor purity and low levels of ICPs and HLA genes, indicating that the poor prognosis in the high-risk group was associated with immune escape. Furthermore, the high-risk group exhibited a notable abundance of M0, M1, and M2 macrophages. As per a prior study, tumor-associated macrophages (TAMs) could potentially contribute to tumor aggressiveness and immunosuppression, ultimately leading to an unfavorable outcome for CC [62]. The GSEA results demonstrated that the DEGs were highly enriched in the differentiation of Th1 and Th2 cells, further supporting this point. A previous report also noted an imbalance in Th1/Th2 cell differentiation in CC and implied that a shift toward Th2 responses could potentially contribute to immune evasion and tumor progression [63,64]. Therefore, exploring potential targets within immune-related pathways presents a promising avenue for enhancing the immunotherapeutic response in CC.

To unravel the mechanism of miR-484 in HeLa cells, an in vitro cell viability assay was conducted using MTT. The results illustrated that miR-484 overexpression significantly reduced the yellow tetrazolium compound to purple formazan crystals, indicating increased metabolic activity and cancer cell viability. Transfected with miR-484 mimics significantly increased the percentage of HeLa cell viability. Furthermore, miR-484-transfected cells, along with PLME treatment, exhibited an increase in cell viability, mitigating the adverse effects of PLME to some extent. Therefore, by reinstating miR-484 expression through mimic transfection, our study demonstrates that miR-484 accelerates HeLa cell growth and counters the deleterious effects of PLME treatment by promoting proliferation.

Therefore, this study investigates CC, emphasizing the potential of miR-484 as a diagnostic and prognostic marker. The dysregulation of miR-484 in various tumors suggests its significance in cancer biology. The study reveals elevated miR-484 expression in CC and explores its involvement in oncogenic pathways such as Ras signaling. Additionally, it uncovers correlations between miR-484 levels and immune cell infiltration in CC, suggesting tailored immunotherapy approaches. This study also indicates potential benefits of immunotherapies targeting immune checkpoint proteins for patients with elevated miR-484 levels. The investigation of genomic alterations and identification of prognostic genes further enhances our understanding of CC. In vitro experiments demonstrate that miR-484 overexpression promotes viability of HeLa cancer cells and mitigates treatment effects. These findings contribute to our understanding of miR-484’s role in CC progression and suggest potential therapeutic strategies involving PLME. Overall, this study comprehensively elucidates the role of miR-484 in CC, providing insights into personalized treatment strategies and avenues for further research.

## 5. Conclusions

By integrating data from bulk transcriptomic and single-cell analyses, particularly concerning immunotherapy, this study comprehensively demonstrated the prognostic significance and carcinogenic impact of miR-484 in CC. In addition, choosing miR-484 unveiled that its overexpression counteracts the impact of PLME, fostering HeLa cell viability. This affirms the contrasting effect of downregulated miR-484 in proliferation upon PLME treatment. However, the miR-484 expression and its associated prognostic genes were only explored at the cellular level. To gain a more detailed understanding of the specific mechanisms of miR-484 and its relationship with prognostic genes, further in vitro/in vivo experiments are required. These future investigations will provide valuable insights into the clinical application of miRNA-targeted therapy and enhance the prognosis of patients with CC.

## Figures and Tables

**Table 1:** PCR primers used in this study.

Primer Name	Primer Type	Primer Sequence (5′-3′)
FGFR3	Forward	AGGAGCTCTTCAAGCTGCTG
	Reverse	AGGTCCAGGTACTCGTCGG
SCNN1B	Forward	TGCTACCCAGGCATTGACAG
	Reverse	CAGGGCTCAGCTCCGAATAG
CALML5	Forward	GACGGCGAAATCAGCTTCCA
	Reverse	AACTCCTCGTAGTTCACCCG
GAPDH	Forward	CGAAGGTGGAGTCAACGGATTT
	Reverse	ATGGGTGGAATCATATTGGAAC

## Data Availability

The data used to support the findings of this study are included within the article.

## Supplementary Materials

The following supporting information can be downloaded at: https://www.mdpi.com/article/10.3390/biomedicines12040909/s1, Supplementary Figure S1. Relationships between miR-484 expression and clinicopathological parameters in CC. (A) Age ≤ 50 and age > 50. (B) Grade: G1–G2 and G3–G4. (C) Stage I/I/III/IV. (D) With and without radiation. (E–G) TNM stage. Supplementary Figure S2. The protein–protein interaction (PPI) network presents NFs and miRNAs interacting with miR-484-related prognostic genes screened by the Kaplan–Meier analysis. (A) The interaction network of prognostic genes with miRNAs. (B) The interaction network of prognostic genes with NFs.
