# Supplementary material for: Exploring MiR-484 Regulation by Polyalthia longifolia: A Promising Biomarker and Therapeutic Target in Cervical Cancer through Integrated Bioinformatics and an In Vitro Analysis
Source: Biomedicines. 2024 Apr 19;12(4):909. doi: 10.3390/biomedicines12040909 (PMC11047986; doi:10.3390/biomedicines12040909)
Supplement: Supplementary file 1 [file biomedicines-12-00909-s001.zip › biomedicines-2934217-supplementary.pdf]

Supplementary Figure S1. Relationships between miR-484 expression and clinicopathological parameters in 283 CC tissues. (A) Age≤50 and age >50. (B) Grade: G1- G2 and G3-G4. (C) Stage I/I/III/ IV. (D) With and without radiation. (E-G) TNM stage.

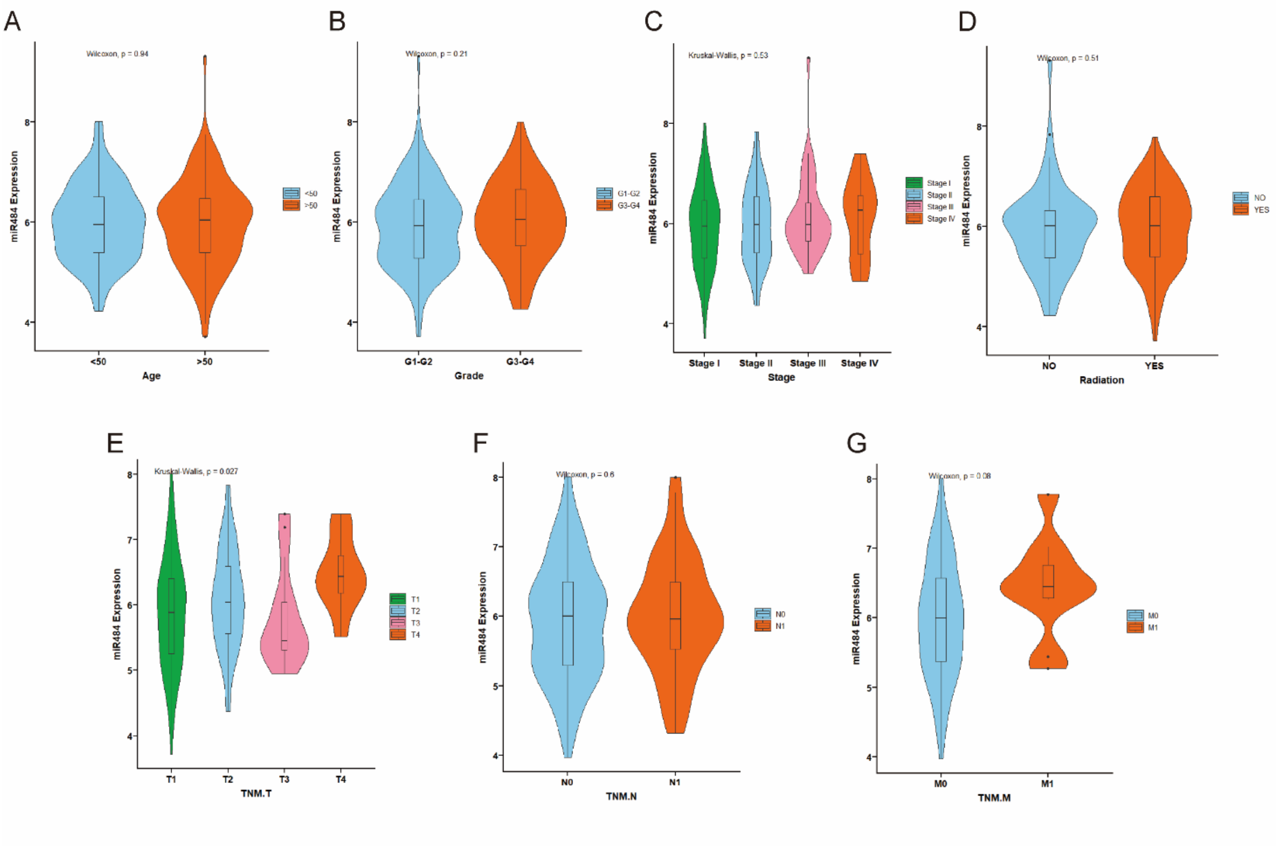

Supplementary Figure S2. The protein-protein interaction (PPI) network presents NFs and miRNAs interacting with miR-484-related prognostic genes screened by Kaplan-Meier analysis. (A) The interaction network of prognostic genes with miRNAs. (B) The interaction network of prognostic genes with NFs.

(A)

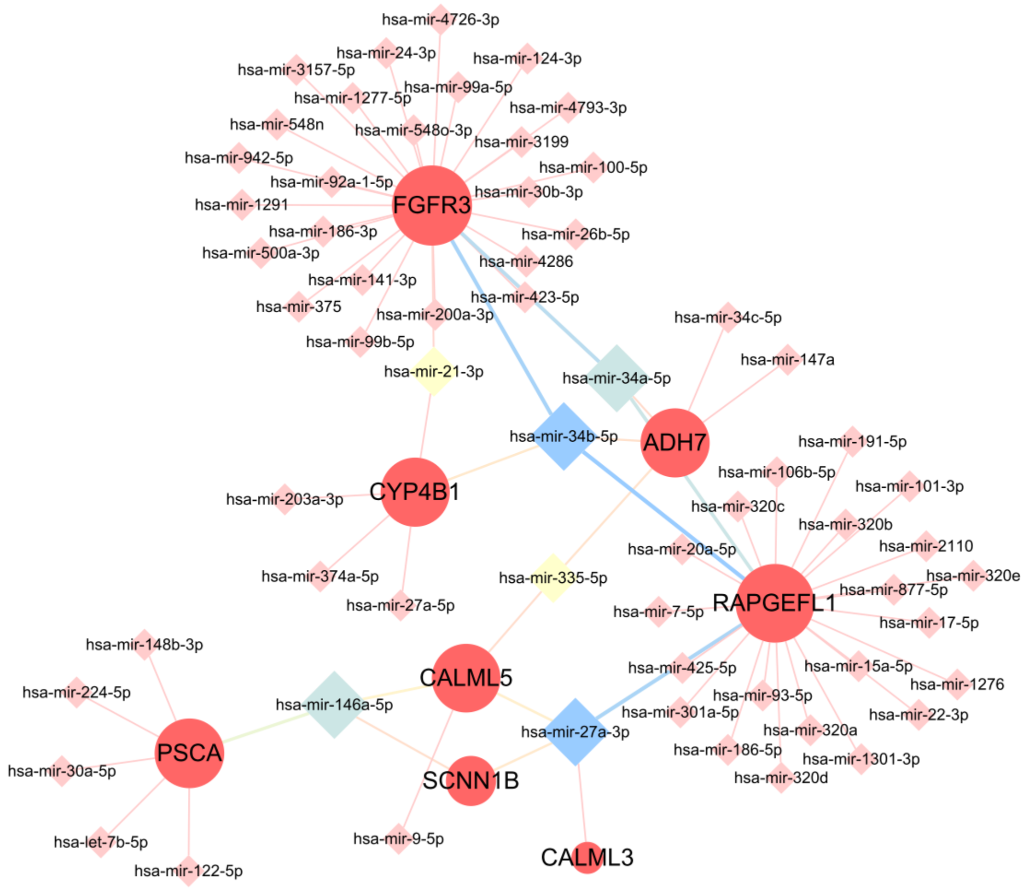

(B)

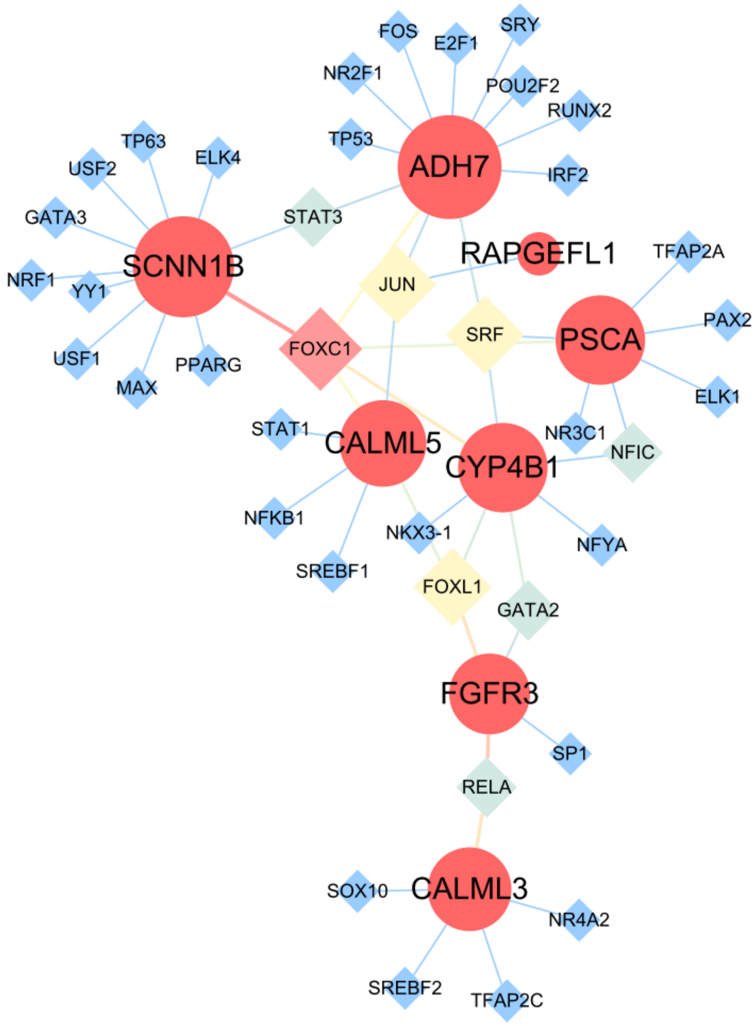
FOS
E2F1
SRY
NR2F1
POU2F2
RUNX2
TP53
ADH7
IRF2
TP63
USF2
ELK4
GATA3
STAT3
SCNN1B
RAPGEFL1
TFAP2A
NRF1
YY1
JUN
SRF
PSCA
PAX2
FOXC1
USF1
MAX
PPARG
ELK1
STAT1
CALML5
NR3C1
CYP4B1
NFIC
NFKB1
NKX3-1
NFYA
SREBF1
FOXL1
GATA2
FGFR3
SP1
RELA
SOX10
CALML3
NR4A2
SREBF2
TFAP2C
